# Iterative Bragg peak removal on X-ray absorption spectra with automatic intensity correction

**DOI:** 10.1107/S1600577524002327

**Published:** 2024-04-09

**Authors:** Ryuichi Shimogawa, Nicholas Marcella, Christopher R. O’Connor, Taek-Seung Kim, Christian Reece, Igor Lubomirsky, Anatoly I. Frenkel

**Affiliations:** a Department of Materials Science and Chemical Engineering, Stony Brook University, Stony Brook, NY 11794, USA; b Mitsubishi Chemical Corporation, Science and Innovation Center, 1000 Kamoshida-cho, Aoba-ku, Yokohama 227-8502, Japan; c Department of Chemistry, University of Illinois, Urbana, IL 61801, USA; d Harvard University, Rowland Institute at Harvard, Cambridge, MA 02142, USA; e Department of Molecular Chemistry and Materials Science, Weizmann Institute of Science, Rehovot 761001, Israel; f Chemistry Division, Brookhaven National Laboratory, Upton, NY 11973, USA; University of Turin, Italy

**Keywords:** X-ray absorption spectroscopy, Bragg peak removal, X-ray absorption coefficient, Bragg peak, crystalline materials

## Abstract

This study introduces a novel iterative Bragg peak removal with automatic intensity correction (IBR-AIC) methodology for X-ray absorption spectroscopy (XAS), specifically addressing the challenge of Bragg peak interference in the analysis of crystalline materials. The approach integrates experimental adjustments and sophisticated post-processing, including an iterative algorithm for robust calculation of the scaling factor of the absorption coefficients and efficient elimination of the Bragg peaks, a common obstacle in accurately interpreting XAS data, particularly in crystalline samples. The method was thoroughly evaluated on dilute catalysts and thin films, with fluorescence mode and large-angle rotation. The results underscore the technique’s effectiveness, adaptability and substantial potential in improving the precision of XAS data analysis. While demonstrating significant promise, the method does have limitations related to signal-to-noise ratio sensitivity and the necessity for meticulous angle selection during experimentation. Overall, IBR-AIC represents a significant advancement in XAS, offering a pragmatic solution to Bragg peak contamination challenges, thereby expanding the applications of XAS in understanding complex materials under diverse experimental conditions.

## Abstract 2

This study presents iterative Bragg peak removal with automatic intensity correction (IBR-AIC) for X-ray absorption spectroscopy (XAS), a new method targeting Bragg peak interference in the analysis of crystalline materials. Merging experimental techniques with sophisticated post-processing, which includes an iterative algorithm for scaling absorption coefficients and eliminating Bragg peaks, this approach demonstrates significant promise in improving the quality of XAS data for these materials.

## 1. Introduction

One of many legacies left by Carlo Lamberti in the multifaceted field of functional materials is his appreciation that any single analytical technique is inherently limited in its ability to characterize all the relevant functional descriptors of a working material or device. For several decades, he and his collaborators explored the combination of X-ray absorption spectroscopy (XAS) with X-ray diffraction to capture the structural changes at the local scale and in the long range, respectively (Giannini *et al.*, 2020 ▸; Bugaev *et al.*, 2017 ▸, 2018 ▸; Agostini *et al.*, 2010 ▸; Lamberti *et al.*, 2003 ▸; Palomino *et al.*, 2000 ▸). Using extended X-ray absorption fine structure (EXAFS), for example, one can study the local structure around species of interest *in situ*, in a variety of conditions and real-time processes, including operando conditions. X-ray diffraction adds useful information about the average structure and phase composition, via analysis of position and intensity of Bragg peaks.

Ironically, despite their value for the combined studies, Bragg peaks, inherently associated with crystalline samples, often pose a significant challenge to X-ray absorption fine structure (XAFS) studies. These peaks are typically present in samples with crystalline structures, such as thin films grown on crystalline substrates (Lowndes *et al.*, 1996 ▸), metal-organic frameworks (MOFs) (Furukawa *et al.*, 2013 ▸), layered materials (Butler *et al.*, 2013 ▸), zeolites (Zhang *et al.*, 2023 ▸), ferroelectric and piezoelectric materials (Pinto *et al.*, 2022 ▸), superconducting materials (Iida *et al.*, 2023 ▸), *etc*. Their appearance is not just a nuisance; similar to monochromator glitches (Bridges *et al.*, 1992 ▸; Li *et al.*, 1994 ▸; Bauchspiess & Crozier, 1984 ▸), they often contaminate the spectra in the X-ray absorption near-edge structure (XANES) and EXAFS region, reducing the amount of information that can be extracted from the data, and complicate their analysis and interpretation, thereby obscuring the true nature of the material under study. This can be problematic for all the aforementioned materials, but it is particularly challenging for thin films or dilute materials on crystalline substrates, where the XAFS signals can be easily overwhelmed by the Bragg peaks.

To navigate this challenge, various strategies have been developed, both experimentally and in post-analysis. Experimentally, approaches such as grazing-incidence X-ray absorption spectroscopy (Heald *et al.*, 1984 ▸), ones using large divergent X-rays (Chen *et al.*, 2013 ▸), changing the crystallinity of the support or window (Ishimatsu *et al.*, 2012 ▸), spinning stages (Harris, 1997 ▸), or total electron yield measurements (Erbil *et al.*, 1988 ▸) have been employed. These methods, while effective, often require special experimental setups and can conflict with other experimental parameters, especially in *in situ* studies. Post-analysis techniques, on the other hand, are manually carried out by visual examination of an expert or glitch rejection by tolerance methods which are readily available on common EXAFS analysis software (Newville, 2013 ▸; Ravel & Newville, 2005 ▸), or general glitch removal algorithms based on detection of outliers within a moving window (Wallace *et al.*, 2021 ▸). However, these approaches are usually suitable for glitches that have a sharp peak shape, and the user’s experience or hyperparameters will have a strong influence when processing the broad signals that are usually seen in Bragg peaks.

A notable advancement in this area was a method combined with simple experimental manipulation combined with post-analysis (Hong *et al.*, 2009 ▸). This method involved collecting XAFS data at various angles to shift the peak position of the Bragg peaks, followed by a reconstruction of the spectrum from these multiple datasets. This technique proved effective in removing Bragg peaks without relying on assumptions or prior knowledge about the true spectrum, making it a robust solution. Its compatibility with many synchrotron beamlines, which are typically equipped with rotation stages, further enhances its practicality. However, this approach is limited to small-angle rotations and is applicable to transmission mode only (therefore requiring sufficiently large concentrations of the species of interest), because the use of fluorescence mode or large-angle rotation can lead to complex changes in the absolute intensity of the X-ray absorption coefficient.

We hereby report an extension of this method to a large class of systems (*e.g.* dilute) and experimental conditions, including fluorescence. The method, which we call iterative Bragg peak removal with automatic intensity correction (IBR-AIC), relies on both the sample rotation during the experiment and the post-processing for removing Bragg peaks from XAFS data. The optimization of the scaling factor to correct the absolute intensity of the X-ray absorption coefficient and the update method of updating the Bragg peak information was essential for application to the fluorescence mode and large-angle rotation. This method will broaden the scope and utility of XAFS for crystalline materials measurements and *in situ* studies, where the Bragg peaks are unavoidable, without the need for special experimental setups.

In the remainder of this article, we describe the experimental setup, the samples we use to demonstrate this method, the algorithm, and its application to the experimental systems, followed by the conclusions and outlook.

## 2. Methodology

### 2.1. XAS

XAS measurements were performed at the QAS beamline (7-BM) of National Synchrotron Light Source II (NSLS II), Brookhaven National Laboratory, USA. The fluorescence signals were collected using a Canberra PIPS detector equipped at the beamline. The distance from the sample to the PIPS detector was adjusted prior to the measurement to balance the signal intensity and the solid angle of the detector, which affects the peak shape of the Bragg peaks. Pt *L*_3_-edge spectra were collected in fluorescence mode with a 3 µm Zn filter for Pt/SiO_2_ powder and in transmission mode for Pt/SiO_2_ pellets. 0.72 wt% Pt/SiO_2_ was synthesized via a conventional polyol method according to the literature (Kim *et al.*, 2024 ▸). The Pt/SiO_2_ powder was fixed to the aluminium plate by pressurizing the Pt/SiO_2_ sample with the aluminium plate using a hydraulic press. The Pt/SiO_2_ pellet was prepared by pressurizing a Pt/SiO_2_ powder in a 7 mm die using a hand pelletizer. Both Pt samples were mounted to a Nashner-Adler cell (Nashner *et al.*, 1997 ▸) and measured at 350°C in N_2_, 350°C 5% H_2_ in N_2_, the ambient temperature in N_2_, and then the ambient temperature in 0.6% CO in He. For each gas and temperature condition, X-ray absorption spectra were collected for sample angles of 30°, 35°, 40°, and 45°, with respect to the incident beam. The fluorescence detector was located at 90° with respect to the incident beam. The Y *K*-edge was measured in fluorescence mode for Al_0.25_Y_0.75_N thin film (2 µm) grown on an Si substrate (AlYN), prepared according to the literature (Cohen *et al.*, 2024 ▸). The X-ray absorption spectra of AlYN were collected for sample angles of 25°, 30°, 35°, 40°, 45°, and 50°, with respect to the incident beam. The fluorescence detector was located at 90° with respect to the incident beam.

### 2.2. Algorithm

**Figure 1:**
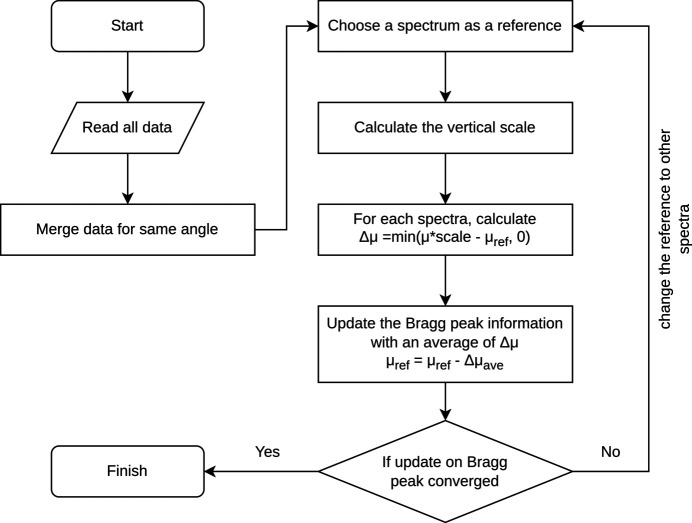
Flow chart for the iterative algorithm method for Bragg peak removal.

An iterative method was applied to the X-ray absorption spectra collected at different angles. The main flow of the algorithm is shown in Fig. 1 ▸. The main component of the iterative algorithm consists of three parts: (i) for any two spectra, scaling all the data vertically, as needed, for their absorption coefficient trend lines (pre-edge and post-edge) to match; (ii) isolation of the Bragg peak contributions by taking difference spectra; (iii) removing the Bragg peak based on the difference spectra. Preprocessing (reading the data and merging the data with the same angle) and postprocessing [usual EXAFS analysis performed by *DEMETER* (Ravel & Newville, 2005 ▸), *Larch* (Newville, 2013 ▸) or any other packages] are also required but are not the main context of the algorithm.

The scaling step (i) is required when the angle or rotation is relatively large, causing the intensity of the absorption coefficient to change with respect to the rotation of the sample. This is required for both fluorescence and transmission mode. For conventional EXAFS analysis, this scaling factor is calculated during the normalization process as an edge step, where the pre- and post-edge processing or the MBACK algorithm (Lee *et al.*, 2009 ▸; Weng *et al.*, 2005 ▸) are used. However, these methods could not be used for the spectra contaminated with strong signals of the Bragg peak or any kind of glitches to estimate the edge step in a reliable manner. This is because the optimization process is done by least-square methods, which can easily be biased by highly intense outliers. To overcome this issue, in our method the scaling factors *c*_*i*_ were calculated by minimizing the mean square root error (MSRE) between scaled absorption coefficients and the reference spectrum [equation (1)],

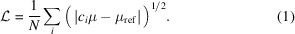

**Figure 2:**
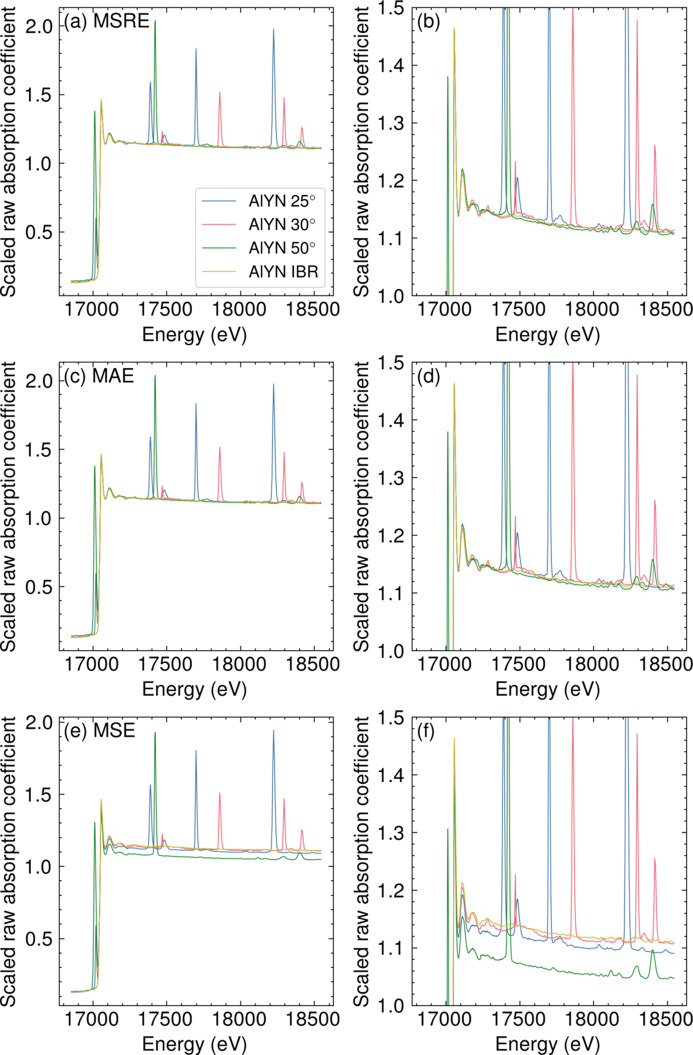
The effect of the objective functions in the calculation of the scaling factors. AlYN spectra at the Y *K*-edge scaled by coefficients obtained by the objective function of (*a*, *b*) MSRE, (*c*, *d*) MAE, and (*e*, *f*) MSE. The figures in the right-hand column show the regions of the post-edge absorption coefficients in greater detail than the corresponding left-hand-column figures.

Employing the MSRE as the objective function, 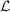 is essential for reliably determining the scaling factor by minimizing the bias arising from the intense signals of Bragg peaks. Compared with the mean absolute error (MAE) or mean square error (MSE), the MSRE tends to underestimate the impact of high-intensity outliers. This feature of using MSRE as a loss function has significant influence in calculating the vertical scale compared with MAE or MSE, as demonstrated in Fig. 2 ▸. The objective function of MSRE [Figs. 2 ▸(*a*) and 2(*b*)] outperformed the scale calculated by MSE [Figs. 2 ▸(*e*) and 2(*f*)], giving a robust scaling coefficient even in the presence of large Bragg peaks. MAE [Figs. 2 ▸(*c*) and 2(*d*)] was nearly equal to the results obtained by MSRE. However, we choose to use MSRE since the objective function becomes less sensitive to large outliers when the power decreases.

While the idea of using an iterative method was first proposed by Hong *et al.* (2009 ▸), our algorithm for detecting and calculating the contributions of the Bragg peaks to EXAFS is different. Our method calculates the contribution of the Bragg peak by calculating any signal that has a positive intensity compared with other spectra after the scaling, while the Hong *et al.* method relies on iterative glitch detection based on the threshold and comparison with the average spectra. Our method has the following advantages. First, Bragg peaks are always positive, and we use this information in the algorithm to improve the detection. Second, this method preserves the information of the relative intensity of the Bragg peak in individual spectra. For example, if we take the difference between a given spectrum (after scaling) and a reference spectrum, the Bragg peaks will appear as positive intensity features, and Bragg peaks in the reference spectrum will appear as negative intensity features. Therefore, updating the contribution of the Bragg peaks is straightforward in individual spectra compared with average spectra.

The algorithm for updating the Bragg peaks is also designed in an iterative manner to take care of the effect of the Bragg peaks on the scaling factor. Ideally, if the scaling factors are calculated correctly, the information on the Bragg peaks can be obtained in a single iteration from the difference spectrum. However, the initial spectra are always contaminated by Bragg peaks, and therefore the scaling factors will be affected, giving offsets to the spectrum. This offset will lead to misalignment in the difference spectrum and introduce an artifact to the information of Bragg peaks that can be obtained from the difference spectrum. In order to reduce the effect of the artifact from the misalignment in the vertical scale of the spectra, the correction of the spectra to remove the Bragg peaks is updated by a small fraction per single iteration that will converge within a significant amount of the iteration. In the algorithm, the amount of the fraction to update the spectra is defined by the maximum intensity of the difference spectrum divided by the number of spectra. This method will ensure that the calculation of the scaling factor will gradually converge to the correct value along with the correction to the spectra for removing the Bragg peaks.

### 2.3. Software

The software is written in Python 3.11 using *NumPy* (Harris *et al.*, 2020 ▸) and *SciPy* (Virtanen *et al.*, 2020 ▸) and made public through GitHub under an MIT license (Shimogawa *et al.*, 2024 ▸). While the module’s input is a *NumPy* array of energy and absorption coefficients, the module can also accept *Larch* groups as input, a common package for handling EXAFS in the Python interface (Newville, 2013 ▸).

## 3. Results and discussion

Two examples of the algorithm will be demonstrated, one for heterogeneous catalysts and one for thin films. The first example will be a mock example of measuring a dilute catalyst dominated by large Bragg peaks, with a comparison with a well prepared pellet as a reference. From this result, we will validate our method and find the limitations of the algorithm. The second example will be the application of thin films, which will be an example of a highly concentrated thin film, dominated by large Bragg peaks from the substrate.

### 3.1. Validation and limitation of the algorithm

**Figure 3:**
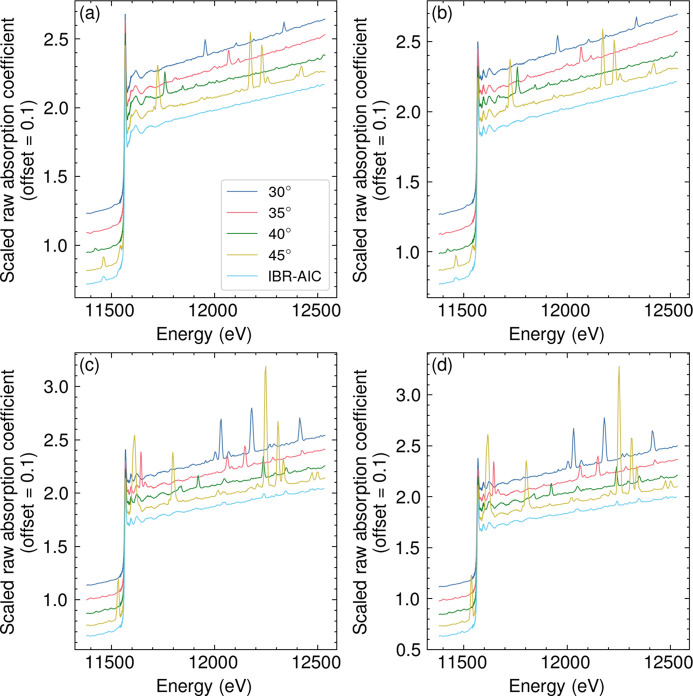
Raw Pt *L*_3_-edge XAFS of Pt/SiO_2_ obtained under different *in situ* conditions: *(a*) 350°C in N_2_, (*b*) 350°C 5% H_2_ in N_2_, (*c*) ambient temperature in N_2_, and (*d*) ambient temperature in 0.6% CO. For each condition, sample angles of 30°, 35°, 40°, and 45° were measured and plotted with the result of iterative Bragg peak removal (IBR-AIC).

**Figure 4:**
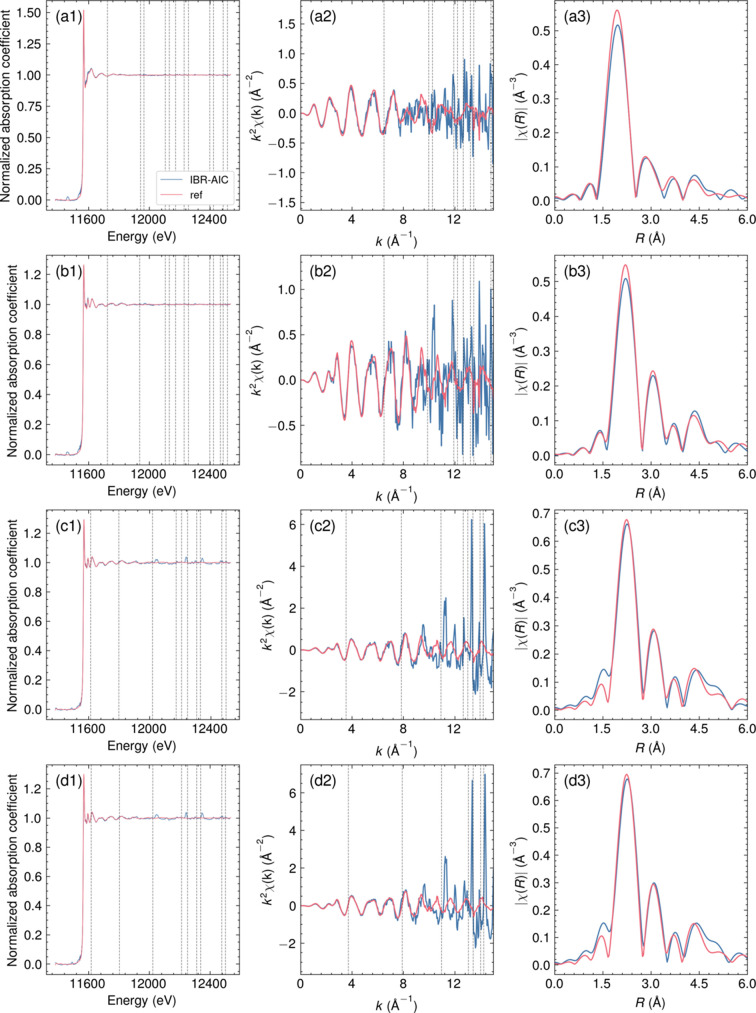
Comparison of the IBR-AIC data of the Pt/SiO_2_ on Al and the transmission signals obtained from the Pt/SiO_2_ pellet under different *in situ* conditions: (*a*) 350°C in N_2_, (*b*) 350°C at 5% H_2_ in N_2_, (*c*) ambient temperature in N_2_, and (*d*) ambient temperature in 0.6% CO. The data are shown in (1) energy space, (2) *k* ^2^-weighted EXAFS spectra, and (3) Fourier transform magnitude of the *k* ^2^-weighted EXAFS spectra. The *k*-range used for the Fourier transforms was from 2 Å^−1^ to 8 Å^−1^. The dashed lines are the positions of the Bragg peaks observed in the datasets.

The validation of the method was demonstrated using Pt/SiO_2_ with two different forms: (i) a powder mounted and pressed on the Al plate as a mock sample of thin films with Bragg peaks (Pt/SiO_2_ on Al) and (ii) a pellet sample suitable for reference transmission measurement (Pt/SiO_2_ pellet). The results of Pt/SiO_2_ on Al measured at different angles scaled by the MSRE objective function are shown in Fig. 3 ▸ along with the results of IBR-AIC, and a comparison of the Pt/SiO_2_ on Al data with the Pt/SiO_2_ pellet data is shown in Fig. 4 ▸. Fig. 3 ▸ clearly shows that the scaling factor of the samples measured at different angles can be reliably calculated using the MSRE as the objective function. While each measurement of Pt/SiO_2_ on Al showed complicated Bragg peaks from the Al plate, the Bragg peaks were efficiently removed by IBR-AIC, as shown in Fig. 3 ▸. The comparison with the Pt/SiO_2_ pellet showed great agreement up until *k* = 10 Å^−1^, while the signals above *k* = 10 Å^−1^ were dominated by high noise. The Fourier transform of the *k* ^2^-weighted EXAFS spectra (Fig. 4 ▸) showed a good match with the reference spectra. A comparison with the conventional manual deglitch is given in Fig. S1 of the supporting information. The manual deglitching also gave a similar result in the low-*R* region but had a slight mismatch in the high-*R* region because of the small Bragg peaks that could not be removed manually.

The three possible causes of the disagreement in the high-*k* region are the following: (i) overlaps in the Bragg peaks throughout the different angles at the high-*k* region, which led to the incomplete removal of Bragg peaks; (ii) the signal-to-noise ratio was not good enough for the dilute sample, compared with the pellet; (iii) the presence of nonlinear energy dependency of the absorption coefficients in the high-energy region. These three causes that were revealed are the potential limitations of our method. To demonstrate the effect of these three factors, we have prepared mock examples by adding some artificial peaks, noise and nonlinear drift to the fluorescence signal obtained in Pt/SiO_2_ at ambient temperature under N_2_ atmosphere (Figs. S2–S8 of the supporting information).

Since our algorithm is technically an intensity comparison of different spectra at each point in the energy grid, there is no way to reconstruct the Bragg-peak-free data when all of the spectra at a specific energy are contaminated with the Bragg peaks. As shown in Fig. S4, the overlap in the Bragg peaks leads to artifacts in the spectrum that we obtain by IBR-AIC.

The limitation applied to the signal-to-noise ratio can be explained by the fact that it is proportional to *n* ^−1/2^, where *n* is the number of samples to be averaged. In the case of the standard XAS measurement, the number of samples will be equivalent to the number of scans measured. On the contrary, the IBR-AIC method decreases in the order of 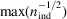 , where *n*_ind_ is the number of scans at individual angles. Figs. S4 and S5 clearly show that if the noise is introduced to one of the spectra it would directly affect the signal-to-noise ratio in the output of the IBR-AIC. The fact that we need separate merging for each angle will require us to collect the same number of scans per angle compared with the conventional merging process, which will multiply the measurement time by the number of angles considered. We also must note that our iterative algorithm will be affected by the spectrum that has the highest signal-to-noise ratio.

In the situation when there is a nonlinear energy dependence our method will fail in that energy region. The nonlinear energy dependence of the absorption coefficients will introduce artifacts (offsets) in the difference spectrum. This nonlinearity can be seen for some of the angles in the high-energy region of Pt/SiO_2_ [Figs. 3 ▸(*a*) and 3(*b*)]. Figs. S7 and S8 demonstrate the effect of the nonlinear response of the absorption coefficients to the output of the IBR-AIC algorithm, where the Bragg peaks were not able to be removed due to the effect of the nonlinear energy dependency. Related to the requirements in the nonlinearity, the analysis is limited to relatively uniform samples, *i.e.* those not causing leakage of X-rays around or through the sample.

To minimize the effect of these limitations, we propose the following tips for the experiment and its post-processing. (i) Collect as many scans as possible, with the same number of scans per angle. (ii) Use the smallest set of angles for which Bragg peaks do not overlap for all spectra. (iii) If the number of data sets is sufficient, omit a set of angles from the input that have a nonlinear energy response or are highly dominated by the Bragg peaks.

### 3.2. Application to thin films

We have further applied the method to a highly concentrated thin film dominated by large Bragg peaks from the substrate. These forms of materials are very interesting to study because it is very common to prepare a thin film on a substrate through epitaxial growth, chemical vapor deposition or any other methods. We used the Y *K*-edge spectra of AlYN (Cohen *et al.*, 2024 ▸) for a test case of our method. AlYN is a potential candidate for compatible piezoelectric materials that has been recently investigated by XAFS (Cohen *et al.*, 2024 ▸).

**Figure 5:**
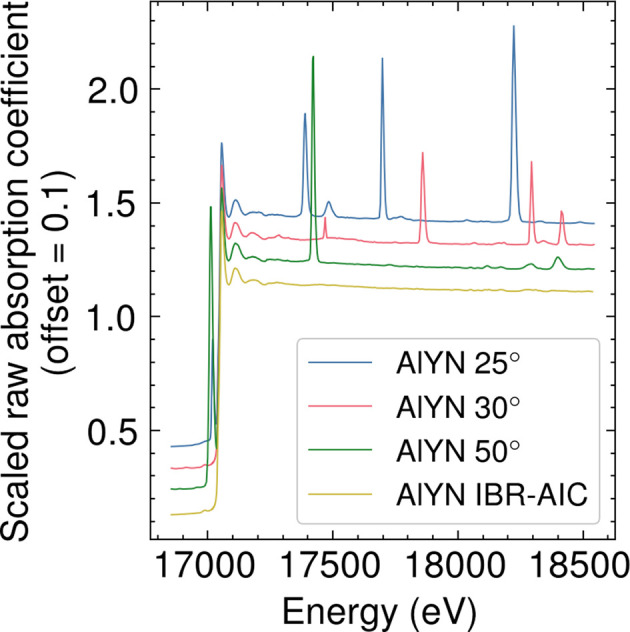
Raw Y *K*-edge XAFS of AlYN obtained for the sample angle of 25°, 30°, 35°, 40°, 45°, and 50° with respect to the incident beam, and the result of iterative Bragg peak removal (IBR-AIC).

**Figure 6:**
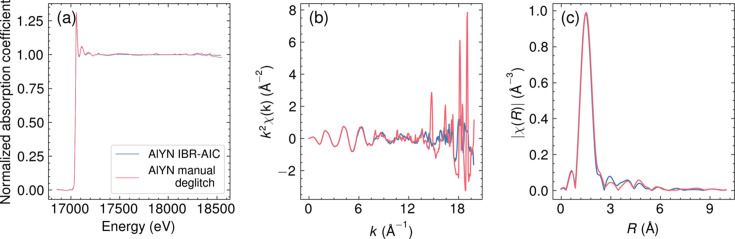
Comparison of the IBR-AIC data with the manually deglitched data of 30° from the literature (Cohen *et al.*, 2024 ▸). The XAS in (*a*) energy space, (*b*) *k* ^2^-weighted EXAFS spectra, and (*c*) Fourier transform magnitude of the *k* ^2^-weighted EXAFS spectra. The *k*-range used for the Fourier transforms was from 2 Å^−1^ to 7.5 Å^−1^.

The results of AlYN measured at different angles scaled by the MSRE objective function are shown in Fig. 5 ▸, along with the results of IBR-AIC, and the comparison of the IBR-AIC method with the manual deglitching from the literature (Cohen *et al.*, 2024 ▸) is shown in Fig. 6 ▸. We did not include all the angles we measured but instead selected the least number of angles that Bragg peaks do not overlap, as discussed in the limitation of the method. Fig. 5 ▸ clearly shows that the scaling factor of the samples measured at different angles is reliable. The spectrum obtained by IBR-AIC showed a good match with the spectrum obtained from the manual deglitching of the spectrum collected at 30° (Fig. 6 ▸), with a slightly noticeable difference in the broad peak around ∼17500 eV. The spectrum collected at a 30° angle has a broad Bragg peak at ∼17500 eV (Fig. 5 ▸), which is not present at other angles. The IBR-AIC method was able to reliably remove the broad peak from the spectra, while it is very dificult to remove these types of peaks manually. It is also noteworthy that the IBR-AIC method worked in the presence of the single-crystal phase of SiO_2_, where SiO_2_ is the substrate of the AlYN, which indicates that the Bragg peaks from the single crystal can equally be treated in the algorithm. The comparison with the manual deglitching showed great agreement until *k* = 14 Å^−1^; however, the removal of the small Bragg peaks is better in the IBR-AIC. The Fourier transform of the *k* ^2^-weighted EXAFS spectra [Fig. 6 ▸(*c*)] showed a good match with the manual deglitching.

## 4. Conclusion

In conclusion, we have presented a method – iterative Bragg peak removal with automatic intensity correction (IBR-AIC) – for the effective removal of Bragg peaks from XAS data, particularly in cases where Bragg peaks pose significant challenges to the analysis. Our approach combines experimental data acquisition with post-processing techniques to eliminate Bragg peaks without the need for specialized experimental setups.

The application of IBR-AIC to various experimental conditions, including fluorescence mode and large-angle rotation, has demonstrated its robustness and versatility. We have demonstrated the method using dilute catalysts and thin films, providing valuable insights into its strengths and limitations.

While IBR-AIC proves effective in most cases, it is important to note the potential limitations associated with signal-to-noise ratios, nonlinear energy dependencies and the need for careful angle selection. Nevertheless, our method offers a valuable tool for researchers in functional materials and X-ray spectroscopy, expanding the scope and utility of XAS for crystalline materials measurements and *in situ* studies. We believe this method will find wide application in materials science research and contribute to a deeper understanding of complex materials and their behavior under various experimental conditions.

## Supplementary Material

Supporting Figures S1 to S9. DOI: 10.1107/S1600577524002327/uc5001sup1.pdf
