# Supplementary material for: Iterative Bragg peak removal on X-ray absorption spectra with automatic intensity correction
Source: J Synchrotron Radiat. 2024 Apr 9;31(Pt 3):456–63. doi: 10.1107/S1600577524002327 (PMC11075706; doi:10.1107/S1600577524002327)
Supplement: Supplementary file 1 [file s-31-00456-sup1.pdf]

JOURNAL OF SYNCHROTRON RADIATION

Volume 31 (2024)

Supporting information for article:

Iterative Bragg Peak Removal on X-ray Absorption Spectra with Automatic Intensity Correction

Anatoly, Ryuichi Shimogawa, Nicholas Marcella, Christopher R. O'Connor, Taek-Seung Kim, Christian Reece, Igor Lubomirsky and Anatoly I. Frenkel

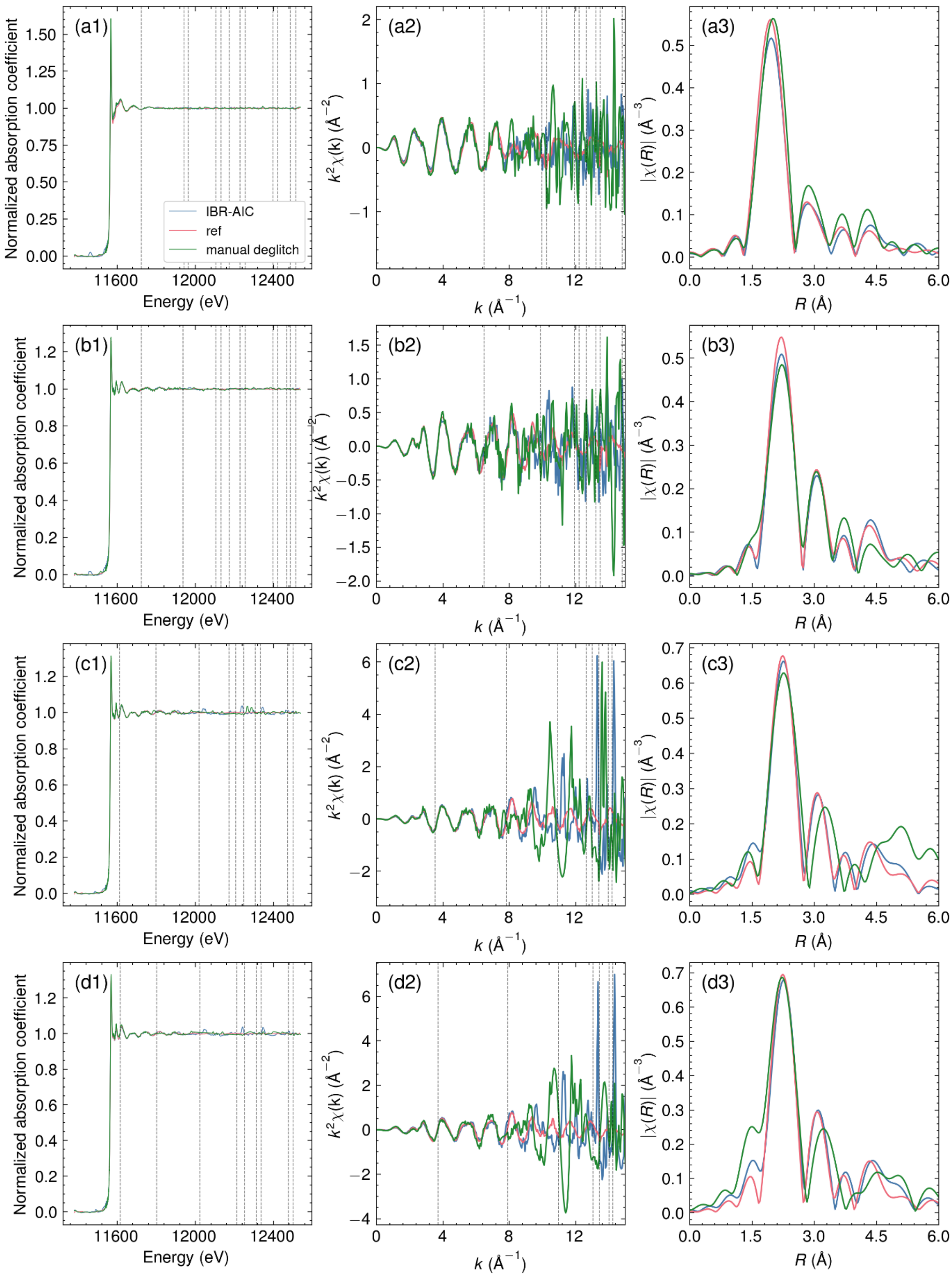

Figure S1 Comparison of the deglitched spectra by IBR-AIC, and manual deglitching of the Pt/SiO_2 on Al and the transmission signals obtained from the Pt/SiO_2 pellet under different in-situ conditions: (a) 350°C at N_2, (b) 350°C at 5% H_2 in N_2, (c) ambient temperature in N_2, and (d) ambient temperature in 0.6 % CO. The data are shown in (1) energy space, (2) k^2-weighted EXAFS spectra, and (3) Fourier transform magnitude of the k^2-weighted EXAFS spectra. The k-range used for the Fourier transforms was from 2 Å^{-1} to 8 Å^{-1}. The dashed lines are the positions of the Bragg peaks observed in the datasets.

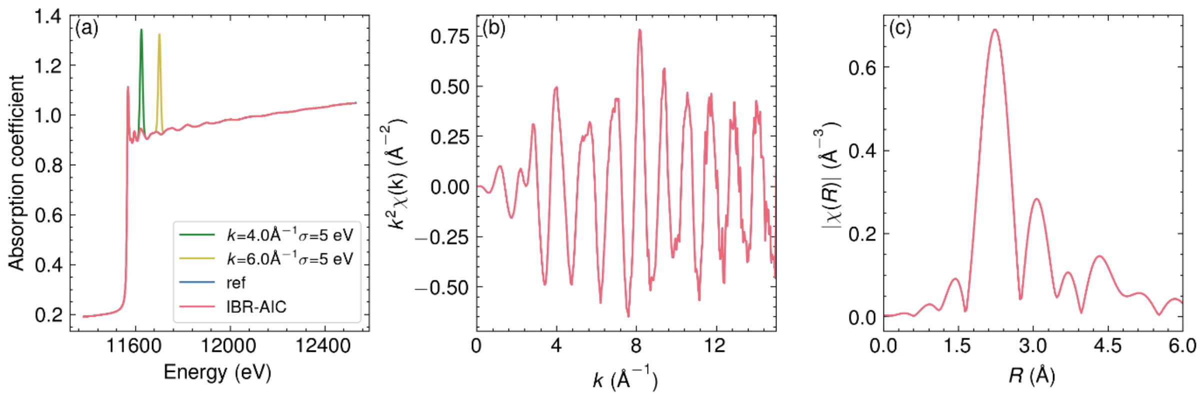

Figure S2 Mock examples of adding artificial Bragg peaks (independent Gaussian peaks at k = 4.0 and 6.0 Å^{-1}, σ = 5 eV) to the fluorescence signal obtained in Pt/SiO_2 at ambient temperature under N_2.. The original spectrum is denoted as ref. The data are shown in (1) energy space, (2) k^2-weighted EXAFS spectra, and (3) Fourier transform magnitude of the k^2-weighted EXAFS spectra. The k-range used for the Fourier transforms was from 2 Å^{-1} to 8 Å^{-1}.

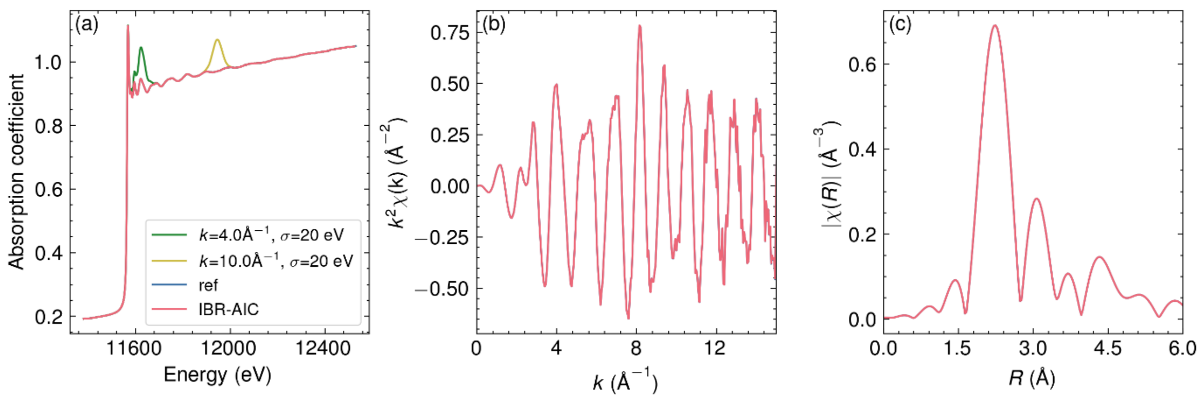

Figure S3 Mock examples of adding artificial Bragg peaks (independent broad Gaussian peaks at k = 4.0 and 10.0 Å^{-1}, σ = 20 eV) to the fluorescence signal obtained in Pt/SiO_2 at ambient temperature under N_2.. The original spectrum is denoted as ref. The data are shown in (1) energy space, (2) k^2-weighted EXAFS spectra, and (3) Fourier transform magnitude of the k^2-weighted EXAFS spectra. The k-range used for the Fourier transforms was from 2 Å^{-1} to 8 Å^{-1}.

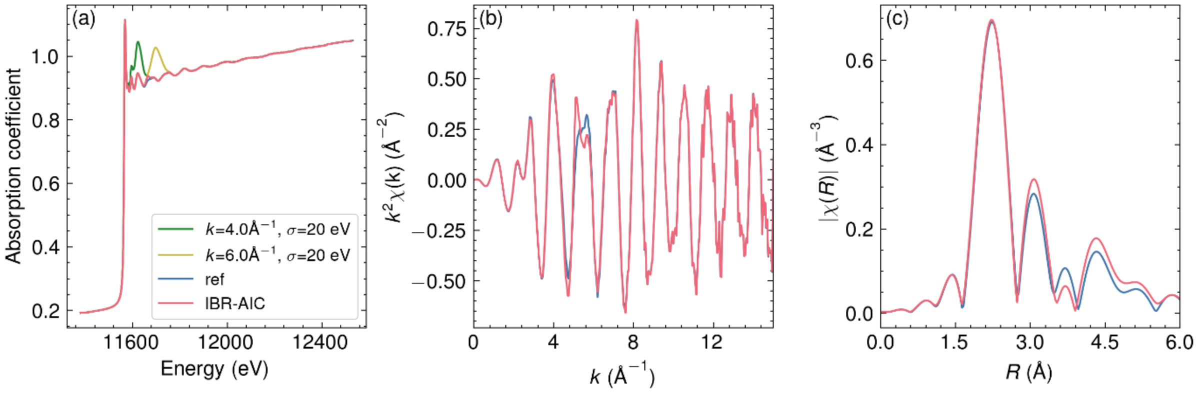

Figure S4 Mock examples of adding artificial Bragg peaks (overlapping broad Gaussian peaks at k = 4.0 and 6.0 Å^{-1}, σ = 20 eV) to the fluorescence signal obtained in Pt/SiO_2 at ambient temperature under N_2.. The original spectrum is denoted as ref. The data are shown in (1) energy space, (2) k^2-weighted EXAFS spectra, and (3) Fourier transform magnitude of the k^2-weighted EXAFS spectra. The k-range used for the Fourier transforms was from 2 Å^{-1} to 8 Å^{-1}.

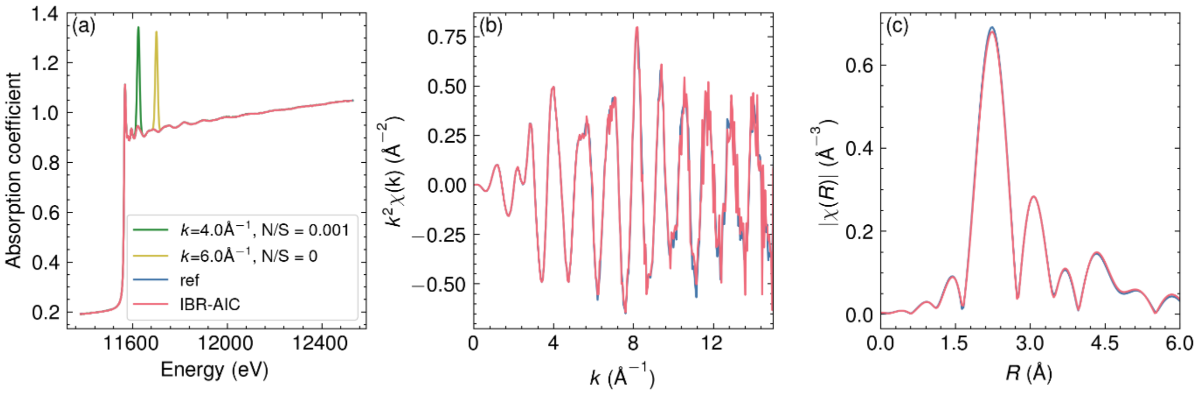

Figure S5 Mock examples of adding artificial noise (Normal distribution with σ = 0.001 × edge step, noise to signal (N/S) ratio of 0.001) to the fluorescence signal obtained in Pt/SiO_2 at ambient temperature under N_2.. The original spectrum is denoted as ref. The data are shown in (1) energy space, (2) k^2-weighted EXAFS spectra, and (3) Fourier transform magnitude of the k^2-weighted EXAFS spectra. The k-range used for the Fourier transforms was from 2 Å^{-1} to 8 Å^{-1}.

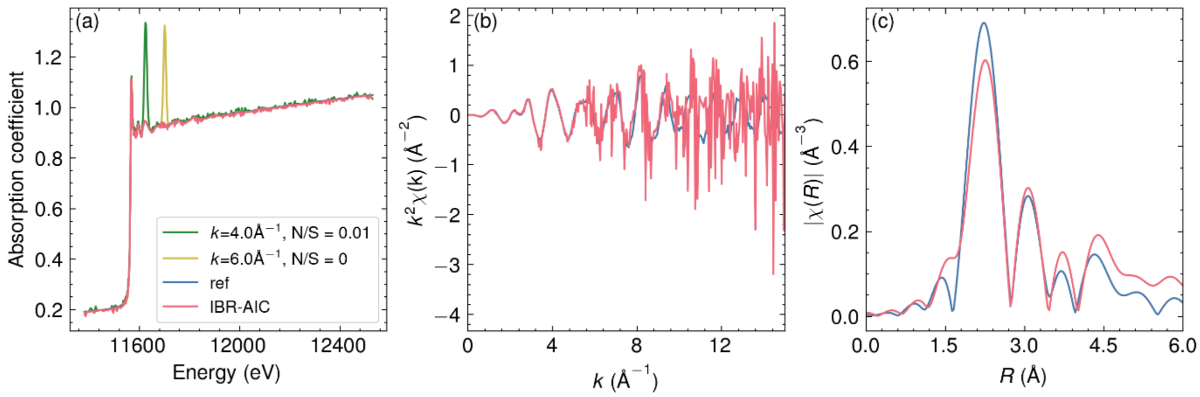

Figure S6 Mock examples of adding artificial noise (Normal distribution with σ = 0.01 × edge step, noise to signal (N/S) ratio of 0.01) to the fluorescence signal obtained in Pt/SiO_2 at ambient temperature under N_2.. The original spectrum is denoted as ref. The data are shown in (1) energy space, (2) k^2-weighted EXAFS spectra, and (3) Fourier transform magnitude of the k^2-weighted EXAFS spectra. The k-range used for the Fourier transforms was from 2 $Å^{-1}$ to 8 $Å^{-1}$.

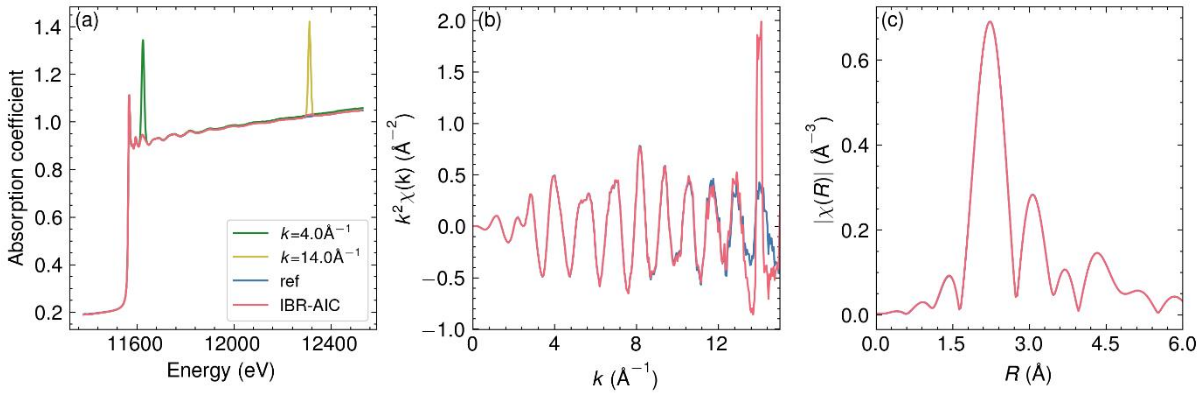

Figure S7 Mock examples of adding artificial non-linearity ($\mu = \mu + 0.0001 \times (\text{Energy} - e0)$) to the fluorescence signal obtained in Pt/SiO_2 at ambient temperature under N_2.. The original spectrum is denoted as ref. The data are shown in (1) energy space, (2) k^2-weighted EXAFS spectra, and (3) Fourier transform magnitude of the k^2-weighted EXAFS spectra. The k-range used for the Fourier transforms was from 2 $Å^{-1}$ to 8 $Å^{-1}$.

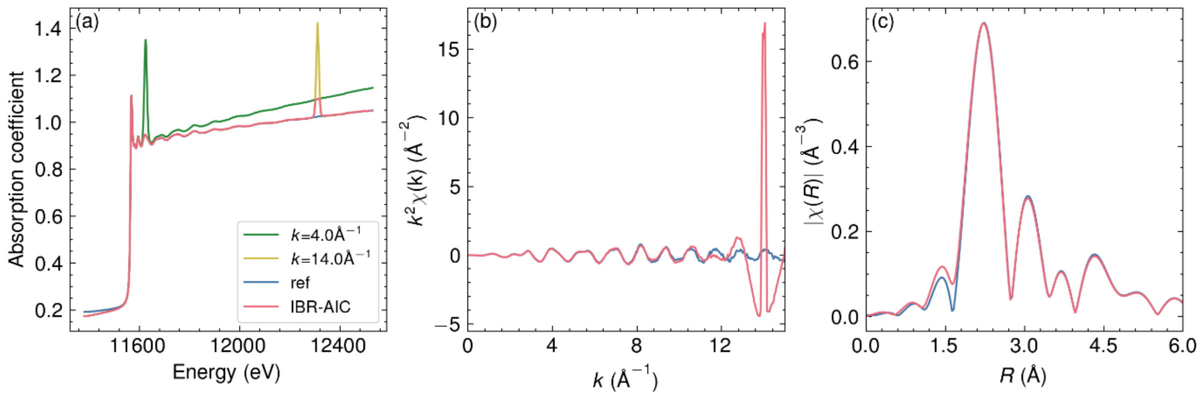

Figure S8 Mock examples of adding artificial non-linearity ($\mu = \mu + 0.001 \times (\text{Energy} - e0)$) to the fluorescence signal obtained in Pt/SiO_2 at ambient temperature under N_2. The original spectrum is denoted as ref. The data are shown in (1) energy space, (2) k^2-weighted EXAFS spectra, and (3) Fourier transform magnitude of the k^2-weighted EXAFS spectra. The k-range used for the Fourier transforms was from 2 $Å^{-1}$ to 8 $Å^{-1}$.

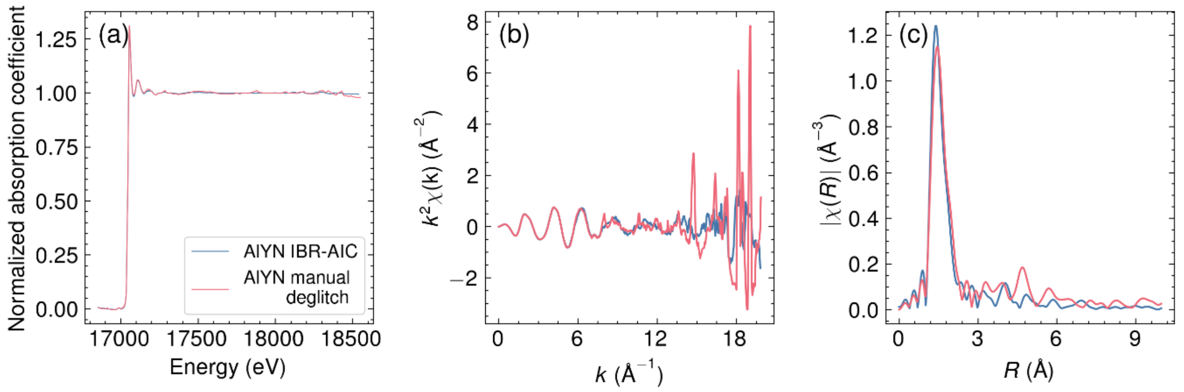

Figure S9 Comparison of the IBR-AIC data with the manually deglitched data of 30° from literature (Cohen *et al.*, 2024). The XAS in (a) energy space, (b) k^2-weighted EXAFS spectra, and (c) Fourier transform magnitude of the k^2-weighted EXAFS spectra.). The k-range used for the Fourier transforms was from 2 $Å^{-1}$ to 12 $Å^{-1}$.

References

Cohen, A., Li, J., Cohen, H., Kaplan-Ashiri, I., Khodorov, S., Wachtel, E., Lubomirsky, I., Frenkel, A. I. & Ehre, D. (2024). *ACS Applied Electronic Materials* DOI:10.1021/acsaelm.3c01390.
